# Evidence for the use of Levomepromazine for symptom control in the palliative care setting: a systematic review

**DOI:** 10.1186/1472-684X-12-2

**Published:** 2013-01-19

**Authors:** Isabel Dietz, Andrea Schmitz, Ingrid Lampey, Christian Schulz

**Affiliations:** 1 Clinic for Anaesthesiology HELIOS Clinic Wuppertal, University Witten/Herdecke, Witten, Germany; 2 Interdisciplinary Center for Palliative Medicine, University Hospital Dusseldorf, Dusseldorf University, Dusseldorf, Germany; 3 NELCS Northeast London (NHS) Community Services, London, United Kingdom

**Keywords:** Levomepromazine, Methotrimeprazine, Palliative care, End of life care, Symptom control, Evidence, Systematic review

## Abstract

**Background:**

Levomepromazine is an antipsychotic drug that is used clinically for a variety of distressing symptoms in palliative and end-of-life care. We undertook a systematic review based on the question “What is the published evidence for the use of levomepromazine in palliative symptom control?”.

**Methods:**

To determine the level of evidence for the use of levomepromazine in palliative symptom control, and to discover gaps in evidence, relevant studies were identified using a detailed, multi-step search strategy. Emerging data was then scrutinized using appropriate assessment tools, and the strength of evidence systematically graded in accordance with the Oxford Centre for Evidence-Based Medicine’s ‘levels of evidence’ tool. The electronic databases Medline, Embase, Cochrane, PsychInfo and Ovid Nursing, together with hand-searching and cross-referencing provided the full research platform on which the review is based.

**Results:**

33 articles including 9 systematic reviews met the inclusion criteria: 15 on palliative sedation, 8 regarding nausea and three on delirium and restlessness, one on pain and six with other foci. The studies varied greatly in both design and sample size. Levels of evidence ranged from level 2b to level 5, with the majority being level 3 (non-randomized, non-consecutive or cohort studies n = 22), with the quality of reporting for the included studies being only low to medium.

**Conclusion:**

Levomepromazine is widely used in palliative care as antipsychotic, anxiolytic, antiemetic and sedative drug. However, the supporting evidence is limited to open series and case reports. Thus prospective randomized trials are needed to support evidence-based guidelines.

## Background

Patients with advanced disease approaching the end of life often suffer from symptoms that impair their own and their families quality of life [1,2]. Alleviation of these symptoms, through a multi-dimensional and inter-professional palliative care approach, includes pharmacotherapy as an essential component.

Common symptoms in the terminal phase are pain, nausea and vomiting, agitation or restlessness and dyspnoea [3]. Pro re nata (PRN) prescription of drugs, as recommended in clinical pathways aim to cover these common symptoms, as well as to provide stand-by medication for possible emergency situations [4,5]. Analgesics, antiemetics, sedatives and anxiolytics titrated to the individual patient’s level of need should be prescribed and any medication, which is not essential for symptom control, discontinued. Drugs administration is preferably via subcutaneous routes, and the amount of patient manipulation related to medication delivery, reduced to a minimum. In severe cases, where patients experience an unbearable and/or refractory symptom burden, palliative sedation therapy may be considered as an important and necessary therapeutic intervention [6,7].

One drug widely used in the palliative care setting is levomepromazine in Europe and methotrimeprazine in the United States (trade names Neurocil, Nozinan, Nosinan or Levoprome). This aliphatic phenothiazine is a neuroleptic with low antipsychotic potency first used in psychiatry for the treatment of schizophrenia [8]. Levomepromazine acts as an antagonist at histamine type 1, muscarinic-cholinergic, dopaminergic 2, alpha-1 adrenoceptor and 5HT-2 receptors [9,10], and due to a half-life of 15–30 hours makes once daily administration practicable. It can be administered subcutaneously, intravenously or orally. Known adverse drug effects include postural hypotension, skin irritation, drowsiness, dry mouth, dystonia, neuroleptic malignant syndrome, Parkinsonism and epilepsy by lowering the seizure threshold [11]–[13]. As a result of the potential impact of some of these side effects on safe mobilisation there are recommendations to best avoid its use in ambulatory palliative care patients [14]. Compared to the cost of some alternative drugs Levomepromazine is a cost effective option (e.g. in the UK 7 tablets with 24 mg of oral levomepromazine costs £1.69; http://www.cks.nhs.uk/) [15]. Table 1 presents the essential pharmacokinetic data of the drug. In palliative care, levomepromazine is predominantly used for the treatment of nausea and vomiting, and for severe delirium or agitation at the end of life. However, its effectiveness is mainly based on anecdotal evidence [16,17]. In clinical practice, its use as a sedative has also become more frequent as part of palliative sedation therapy, and the analgesic properties of levomepromazine are described in some of the studies [18,19]. For most of the above indications the clinical use of levomepromazine is off-label by application in many countries [20] and published evidence is scarce.

**Table 1:** **Pharmacokinetic data of levomepromazine**[21,22]


Bioavailability	20-40% p.o.
Onset of action	30 min.
Maximum serum concentration (t_max_)	p.o.: 2–3 h
	i.m.: 30–60 min
Half life (t_1/2_)	15-30 h
Duration of action	8 h

The use of levomepromazine for symptom control in palliative care has been considered in several published systematic reviews concerning individual symptoms, such as the treatment of nausea and vomiting [23], breathlessness [24] or sedation [25]. However, to date no systematic review has tried to collate the overall evidence base for using this interesting drug in the palliative care setting. The rationale for this investigation is levomepromazine’s broad-range applicability. Potentially, its properties are particularly beneficial in the treatment of several and diverse symptoms in end-of-life care. This review therefore aims to summarise and update the available evidence for the use of the ‘all-rounder’ levomepromazine/methotrimeprazine for patients in the palliative care setting, with a special focus on its utility in symptom control at the end-of-life. The report follows the reporting standard of the PRISMA-Statement. Table 2 presents our research question according to the PICOS approach [26].

**Table 2:** **PICOS approach in our systematic review according to the PRISMA guideline**[26]

	
P	patients	patients at the end of life
I	intervention	pharmacological treatment with levomepromazine
C	comparison	none
O	outcome	symptom control with levomepromazine
S	study design	randomized controlled trials, prospective trials, cohort studies, case series, case reports, systematic reviews

## Methods

A review protocol was developed and the trial was registered with the PROSPERO network for systematic review registration (registration number: CRD42012002390).

### Study characteristics

Publications that met the inclusion criteria were those that 1) involved individuals treated in the palliative care setting, 2) included adults, 3) evaluated pharmacological treatment of symptoms at the end of life with levomepromazine and 4) were characterized as randomized controlled trials, prospective trials, cohort studies, case series or case reports. Systematic reviews were also included but were primarily used for hand searches of references. Non-systematic or narrative reviews were excluded, but collected as a separate category as proof of existing clinical knowledge/practice. Our systematic review was limited to studies published in English or for which English abstracts were available. The period of review was from 1980 to April 2012.

### Search strategy

The following five computerized online databases were searched in the second week of April 2012: Medline (1946 to April week 2 2012), Embase (1980 to 2012 Week 15), The Cochrane Library, PsychInfo (1806 to April week 3 2012), Ovid Nursing (1946 to April week 2 2012).

The automated search was conducted using two main components: The first component included several search terms for identification of literature relevant to palliative care, based on a master search strategy developed for that specific purpose [27], enlarged by some additional search terms. The second component contained the search terms for levomepromazine.

Search terms of the automatic search are the following:exp advance care planning/OR exp attitude to death/OR exp bereavement/OR death/OR hospices/OR life support care/OR palliative care/OR exp terminal care/OR terminally ill/OR palliat*.tw. OR hospice*.tw. OR “terminal care”.tw. OR terminally ill patient.mp. or exp terminally ill patient OR exp terminal care/OR palliat*.tw. OR hospice*.tw. OR end of life care.mp. OR EOL care.mp. OR palliative therapy.mp. or palliative therapy/OR terminally ill patient.mp. or terminally ill patient/AND levomepromazine.mp. or levomepromazine/OR methotrimeprazine.mp. OR neurocil.mp. OR nozinan.mp. OR levoprome.mp.

Table 3 shows the full electronic search strategy as performed in Embase.

**Table 3:** Electronic search strategy performed in Embase

**No.**	**Search term**	**Results**
1	exp advance care planning/	448482
2	exp attitude to death/	8692
3	exp bereavement/	5150
4	death/	92098
5	hospices/	6053
6	life support care/	77969
7	palliative care/	40548
8	terminal care/	20376
9	terminally ill/	5136
10	palliat$.tw.	54515
11	hospice$.tw.	8617
12	terminal care.tw.	1478
13	physician-patient relations/	74874
14	prognosis/	385402
15	quality of life/	193187
16	survival rate/	119505
17	treatment outcomes/	0
18	attitude to health/	70459
19	palliative care.mp.	17299
20	exp terminal care/	40896
21	exp terminally ill patient/	5298
22	terminally ill.mp.	8305
23	exp palliative therapy/	52501
24	EOL care.mp.	307
25	1 or 2 or 3 or 4 or 5 or 6 or 7 or 8 or 9 or 10 or 11 or 12 or 13 or 14 or 15 or 16 or 17 or 18 or 19 or 20 or 21 or 22 or 23 or 24	1405466
26	levomepromazine.mp.	4195
27	levomepromazine/	4103
28	methotrimeprazine.mp.	127
29	methotrimeprazine/	4103
30	neurocil.mp.	206
31	nozinan.mp.	480
32	levoprome.mp.	34
33	26 or 27 or 28 or 29 or 30 or 31 or 32	4223
34	25 and 33	499
35	limit 34 to english language	364
36	limit 35 to yr = “1980 -Current”	363

### Study selection

After conducting the search in all databases and de-duplication, as a first step, titles and abstracts of identified studies were screened for relevance to the topic and studies considered being not relevant excluded. In a second step, full texts were sought for all studies, which appeared to meet the inclusion criteria. Conference abstracts were also included. Two independent researchers then separately reviewed all retrieved papers for relevance. Where a difference in results occurred, data was discussed and the discussion recorded. Final decisions were strictly based on adherence to the inclusion and exclusion-criteria. If agreement could not be reached, full-text analysis using a relevant quality-instrument was performed. Where there was still no agreement after thorough discussion, the study was included into the search and its relevance discussed in the publication.

### Data extraction and assessment of studies

Relevant studies were extracted into a qualitative synthesis table and categorised according to the following items: author, title, year of publication, journal, study design, indication for levomepromazine, study population, setting, number of study participants, number of patients under treatment, mean dose, dose range, application, measurement of effectiveness, reported adverse effects, remarks, conclusion, main results from the quality analysis process, further comments.

Studies were critically appraised and the evidence was graded based on the determinants for quality of evidence published by the Oxford Centre for Evidence-Based Medicine Levels of Evidence: Level 1: evidence from a systematic review of RCT; Level 2: evidence from a RCT; Level 3: evidence from a non-randomized controlled cohort studies Level 4: evidence from case-series or case–control or historically controlled studies Level 5: expert opinion [28]. Quality assessments were undertaken by using quality check-lists adherent to the standards gathered by the EQUATOR network [29].

## Results

### Study selection

**Figure 1:**
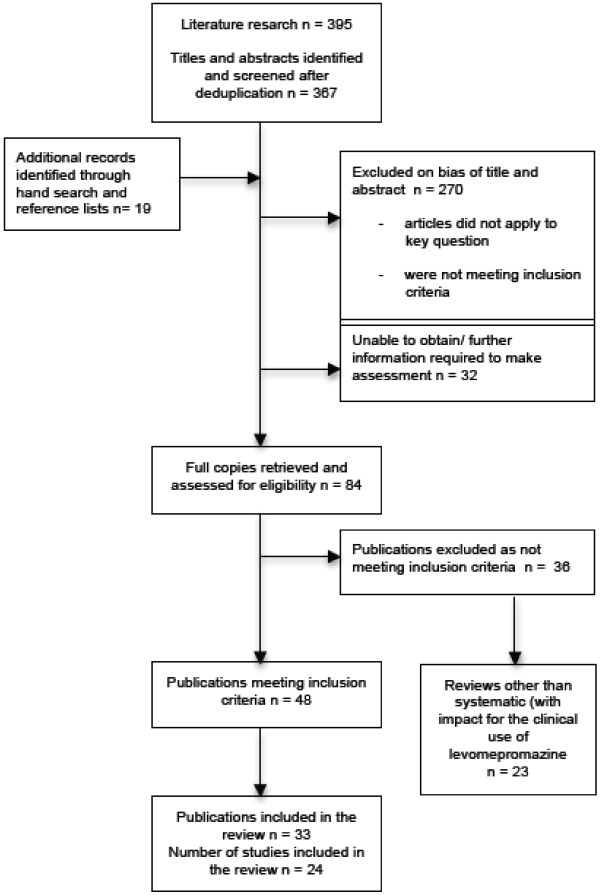
Flowchart of study selection process.

A total of 33 articles involving 9 systematic reviews met the inclusion criteria for research, and reported data regarding patients treated with levomepromazine/methotrimeprazine in a palliative care setting. The search of the five databases initially provided 367 studies after de-duplication. After reviewing of titles and abstracts 270 of these papers were rejected as clearly not meeting the inclusion criteria. 19 additional records were identified through hand-searching and reference lists. Of the remaining 84 references full copies were retrieved and assessed for eligibility. Of those 48 papers met the inclusion criteria. After further examination of these studies in more detail, 33 articles remained for data extraction. 25 out of these 33 papers were found via automatic database searches and eight were found through reference tracking or hand searching. Moreover we identified 23 reviews, other than systematic or narrative, not included in this review, which could be regarded as relevant for clinical practice, and beneficially be analysed in a separate review elsewhere. Figure 1 shows a flowchart of the study selection process.

### Study characteristics

The principal characteristics of the selected articles are presented in the Additional file 1: Appendix. All relevant papers were published in peer-review journals between 1980 and 2011. Seven papers dealt with the topic of sedation, five with nausea and vomiting and one paper each with pain, delirium, several indications and side effects of levomepromazine. Regarding study design, we included six case reports, two survey studies, nine retrospective studies and seven prospective studies. Nine systematic reviews were also included.

### Sedation

The 12 studies and four systematic reviews on palliative sedation presented the largest group within the reviewed articles [30]–[45]. Studies concerning the use of levomepromazine/methotrimeprazine in palliative sedation varied largely in study design and sample size. In a retrospective cohort study of 29 patients sedated at home, two had their medication changed from midazolam to levomepromazine, which was effective in both patients. In one of these patients the indication for sedation was pain, in the other patient delirium was given as the rationale for use [31]. The other studies on sedation did not provide specific information on the background or indication for the use of levomepromazine in palliative sedation. The retrospective chart review of Stone et al. documented levomepromazine in 33 out of 115 reviewed patients (28,7%) in their last 5 days of life [40], Stephenson et al. found that 51–58% of sedated patients received levomepromazine according to their chart review [33]. In a survey study by Chater et al. of 100 patients, reported on by 61 selected palliative care experts, 30 (30%) had received levomepromazine for palliative sedation [39]. Sykes at al showed that levomepromazine was used in only 3 out of 114 patients (2,6%) receiving sedation in a English hospital setting, during their last week of life, but that this number increased to 30 patients (26%) in the last 24 hours [35]. Reutzel et al. asked their respondents in a retrospective survey about one case of end-stage palliative sedation during the past 12 months; in 15 out of 312 reported cases (4,8%) levomepromazine was used [34]. In a multi-center, prospective, observational study on specialized palliative care units in Japan, in a sample of 102 patients the use of levomepromazine was documented in 2 cases (1.9%) [44]. A retrospective chart review of Morita et al. demonstrated the use of levomepromazine for sedation only in 2 out of 209 patients (0,97%) [37] and in a study comparing data from 97 sedated patients in three different countries (Israel, South Africa and Spain), only one received levomepromazine [38].

One of the two case studies presented a patient suffering from motor-neurone disease in which sedation was started to allow withdrawal of mechanical ventilation [30], the other case described sedation due to intractable seizures in a patient with insular thyroid cancer and brain metastases [32].

In most of the cases presented in these studies, levomepromazine was given in combination with midazolam, only Alonso-Babarro changed to levomepromazine alone [31]. Relevant papers for the use of levomepromazine in palliative sedation with recommendations regarding doses and dose range and provided indications for sedation are shown in Table 4.

**Table 4:** Recommended doses and dose ranges for levomepromazine in palliative sedation (data refer to subcutaneous application)

**Author**	**Year**	**Study design**	**Mean dose (mg/24 h)**	**Dose range (mg/24 h)**	**Findings**	
Lebon [30]	2010	case study	25	25-100	levomepromazine in combination with midazolam	
Alonso-Babarro [31]	2010	retrospective cohort study	125	100-150	indications: pain, delirium	
D'Cruz [32]	2009	case report	no data	no data	indication: agitation/delirium	
Stephenson [33]	2008	retrospective chart review	9.4	2.5–75	2006	1996 levomepromazine was often used first line, 2006 midazolam was used first line and levomepromazine as an adjunct
			75	25–150	1996	
Reuzel [34]	2008	survey study	no data	no data	most common indications: pain and dyspnoea	
Sykes [46]	2003	retrospective case–control study	125	125-200	indication: continuing agitation	
Gambles [36]	2001	descriptive retrospective study	no data	6.25-12.5	indication: agitation/restlessness	
Morita E [37]	2001	prospective study	50	50		
Fainsinger [38]	2000	prospective observational multicenter study	no data	no data	most common indication: delirium	
Chater [39]	1998	survey study	100	50–250		
Stone [40]	1997	retrospective chart review	64	no data	levomepromazine was usually prescribed in combination with another sedative	
Oliver ) [41]	1985	retrospective chart review	no data	37.5-300	indications: confusion/agitation, pain, vomiting (not palliative sedation as main indication)	
Mercadante [42]	2011	SR	no data	100-150	based on [39]	
DeGraeff [43]	2007	SR	64	25-250	based on [39][40,45,46]	
Morita T [25]	2005	SR	no data	5-12,5	guideline is based on a Delphi process conducted by the Sedation Guideline Task Force	
Cowan [45]	2001	SR	64	48-600	based on [40,41]	

Effectiveness of sedation was measured in only some of the papers, mostly subjectively rather than with standardized tools [31,38,39,41]. In the work of Alonso-Babarro, effective sedation was achieved with a level 5 or greater on the Ramsay scale and a lack of emergency calls during the process. In three studies survival was measured as main outcome criteria [37,40,46].

### Nausea and vomiting

Papers concerning the use of levomepromazine for nausea and vomiting represented the second largest group: we found six studies [13,47]–[51] and two systematic reviews [23,52] dealing with that topic. Eisenchlas et al. reported on a sample of 70 patients with digestive cancer treated in an open-label prospective study with levomepromazine for nausea and vomiting, in which sixty patients (86%) were categorized as responders. In that study, the Pearson test revealed no association between levomepromazine dose and response to treatment, and no association between levomepromazine dose and degree of sedation [13]. In a quasi-experimental prospective study Kennett et al. showed that levomepromazine is an efficient first line antiemetic in indeterminate patho-physiological causes of nausea and vomiting, and second line for all other causes [50]. These findings were confirmed by a non-comparative prospective study conducted by Stephenson et al. in which, from a sample of 61 hospice patients with nausea, 27 (44%) received levomepromazine [48]. Moreover levomepromazine was proven to be effective as second line treatment in chemotherapy-induced nausea [51] and in carcinoid syndrome [49]. In a survey of 154 oncologists and oncology nurse prescribers levomepromazine was recommended for refractory chemotherapy-related nausea and vomiting as the second or even third-line treatment option [47]. One other paper did not focus on nausea and vomiting as a main issue, but reviewed the extent of drug use for unlicensed purposes in a palliative care unit and found that 8 out of 689 prescriptions (1,2%) were oral levomepromazine for nausea and vomiting, and 18 (2,6%) were subcutaneous levomepromazine [53]. In another systematic review on symptom management for the adult patient dying with advanced chronic kidney disease, levomepromazine was recommended as second line therapy for nausea and vomiting, if haloperidol failed. The authors do not provide doses for levomepromazine in the renal failure population [54].

Table 5 shows recommended doses and dose range of levomepromazine.

**Table 5:** Recommended doses and dose ranges for levomepromazine in nausea and vomiting

**Author**	**Year**	**Study design**	**Mean dose (mg/24 h)**	**Dose range (mg/24 h)**	**Findings**
Molassiotis [47]	2010	survey study	no data	no data	second or third line for refractory chemotherapy-related nausea and vomiting
Stephenson [48]	2006	non-comparative prospective study	no data	6.25–25	first line in indeterminate pathophysiological causes and second line for all other causes of nausea and vomiting
Eisenchlas [13]	2005	open-label prospective study	6.25	3.12- 25	second line
Amesbury [49]	2004	case report	no data	12.5 -25	first line indication: 5HT2 antagonist property of levomepromazine is used because large amounts of circulating 5-HT are present in carcinoid syndrome
Kennett [50]	2004	quasi experimental prospective study	no data	6.25-25	second line or for indeterminate pathophysiological causes
Higi [51]	1980	prospective study	no data	16-30	second line in chemotherapy induced nausea
Davis [23]	2010	SR	nox data	no data	based on [13,49,50]
Glare [52]	2004	SR	no data	no data	based on one study [55] (not available)

### Delirium/terminal restlessness

For the specific use of levomepromazine in delirious or restless patients three papers were included [56]–[58]. One retrospective chart review found that, in 39 hospital patients with delirium during their last week of life, 7 patients (18%) were treated with levomepromazine. A combined treatment of neuroleptics and benzodiazepines was used more often in that study [56]. Fainsinger at al. reported a case of agitated delirium that was treated with levomepromazine after haloperidol and lorazepam had failed. In that case the patient equally failed to respond to the doses of levomepromazine that were used (20–60 mg/24 hours), and the presence of extrapyramidal side effects contributed to the decision to change treatment again, with midazolam the final agent effectively controlling the delirium [58]. That case report is the only paper on levomepromazine included in the systematic review on the treatment of terminal restlessness performed by Kehl et al. 2004, which concluded that there is little empirical evidence suggesting that a single medication or class of medications is superior to another for terminal restlessness [57]. One final paper, which did not focus on delirium as main issue, but was reviewing the extent of drug use for unlicensed purposes in an English palliative care unit, found that 4 out of 689 prescriptions (0,6%) were subcutaneous levomepromazine [53].

### Other indications/issues

The remaining three papers deal with different issues concerning the use of levomepromazine in palliative care. One study focuses on the analgesic quality of the drug. The authors report a case of a patient suffering from pain associated with lung cancer, which was sensitive to opioids and possibly related to bowel shutdown. This patient obtained adequate relief of abdominal pain with a dose of 10 mg levomepromazine i.m.. The authors used a conversion rate of 10 mg levomepromazine to 5 mg of morphine and preferred it because of a smaller effect on the gut and less respiratory depression [59]. Aside from this one case report, no further literature on the analgesic effect of levomepromazine was found. Another case report discusses the possible side effect of levomepromazine-induced lupus erythematosus in a patient with metastatic non-small cell lung cancer [60] and one conference abstract reported the use of levomepromazine in the management of terminal haemorrhage [61]. One systematic review on treatment of intractable breathlessness in patients with advanced cancer showed that there are no randomized controlled trials of phenothiazines in patients with cancer, and that the use of these agents is predicated on evidence in COPD and healthy volunteers. The authors of that review non-the-less recommend the general use of levomepromazine in patients in whom anxiety becomes overwhelming, or for palliative sedation therapy at the end of life [24].

### Effectiveness

Only 12 studies included information about effectiveness or reported information on measurement of effectiveness [13,31,35,37]–[41,48,50,51,58]. In six of these studies, only specific effectiveness of the treatment with levomepromazine was provided, the summarized data is shown in Table 6.

**Table 6:** Studies reporting effectiveness and measurement of effectiveness of levomepromazine

**Indication**	**Reference**	**Number of patients under study**	**Number of patients receiving levomepromazine**	**Effect**
sedation	Alonso-Babarro [31]	29	2	**100%** (defined as symptom control was achieved, consciousness (consciousness was 5 or greater using the Ramsay scale and no emergency calls during the PS process)
nausea	Eisenchlas [13]	70	70	**86%** (categorized as responders if NRS score decreased by 6+ from the baseline score)
nausea	Kennett [50]	65	65	**62.3%** (14 patients met the definition of a complete response and 19 a partial response)
nausea	Higi [51]	113	113	**62%** (fully protected from nausea and vomiting)
nausea	Oliver [41]	675	15	**86%** (no data about how effectiveness was measured)
nausea	Twycross [55]	29	29	**83%** (no data about how effectiveness was measured, study not included in our review)
delirium	Fainsinger [38]	1	1	**0%** (ineffective in treating the patient’s delirium and thus discontinued after 48 hours)
agitation	Oliver [41]	675	49	**67%** (no data about how effectiveness was measured)
pain	Oliver [41]	675	16	**94%** (no data about how effectiveness was measured)

### Assessment of quality and risk of bias

The assessment of quality for the included studies was undertaken according to the standards gathered and regularly updated by the EQUATOR network. Risk of bias was assessed on an individual study level. The PRISMA checklist was used for the nine systematic reviews [26] and in 14 papers quality was assessed using the STROBE check-list for observational studies [62]. The six case reports included in the review were evaluated by the check-list recommended by Sorinola et al. [63], and the four papers reporting survey research were evaluated as suggested by Kelley et al. [64]. No one paper in any category covered all reporting or quality criteria as set out by their corresponding check-list. All survey studies were rated medium to high quality. The case reports averaged medium quality, and the quality of reporting for the systematic reviews and observational studies was only of low to medium quality.

Levels of evidence according to the Oxford Centre for Evidence-Based Medicine Levels of Evidence ranged from level 2b (retrospective/individual cohort study) to level 5 (expert opinion). Most papers (n = 22) were categorized as level 3 (non-randomized, non-consecutive or cohort studies). Only three studies reached level 2: one concerning palliative sedation [31] and two on nausea [13,50]. Details of quality assessments for every study are presented in Additional file 1: Appendix. Further information about the process of quality assessment and use of the check-lists can be obtained from the authors.

As no meta-analysis was conducted and the studies included in our systematic review showed large variations of study design, sample size and quality, no assessment of risk of bias across studies was undertaken.

## Discussion

This review aimed to summarise and update the available evidence for the use of the “all-rounder” levomepromazine/methotrimeprazine for patients in the palliative care setting with a special focus on symptom control at the end of life.

Levomepromazine is a drug with broad-range applicability and effectiveness in the treatment of symptoms in end-of-life care had already been demonstrated in a study by Oliver et al. in 1985 [41]. However, since that study, which looked at the use of this particular drug for confusion and agitation, nausea and vomiting and pain as three main indications for use, no other work has considered levomepromazine in palliative care treatment 'as a whole’. In that early work by Oliver et al., sedation was reported as a noted side effect of levomepromazine, whereas subsequent studies in the 1990^es^ turned that side effect into a benefit and started to realize the value of the drug as a part of treatment where sedation was indicated and/or intended [39,40]. Further researchers began to focus on the use of the drug in specific symptom control for individual symptoms in palliative care patients and an overall perspective on the multifaceted applicability of levomepromazine stepped into the background.

Multiple studies showed that levomepromazine, due to its broad-spectrum action on receptors involved in emesis, is effective as a first-line treatment for intractable patho-physiological causes and as a general second-line option for treatment of nausea and vomiting [13,47]–[51]. Dose ranges vary slightly at the lower value but are stable in the upper; only one study indicated doses up to 30 mg levomepromazine per day, all other studies stated an upper value of no more than 25 mg per day.

There exist a variety of non-systematic reviews and narrative articles recommending levomepromazine for nausea and vomiting in palliative care patients, which should be recognised and considered in practice, although they are mostly based on anecdotal evidence or expert opinion [12,16,56]–[58,65,66]. The two systematic reviews on nausea and vomiting included in our review provide very little information or data on dosage, which leaves them short on clinical applicability [23,52]. However, an expected Cochrane review evaluating the efficacy of levomepromazine for the treatment of nausea and vomiting in palliative care patients may in future, when completed and published, provide some useful guidance towards establishing recommendations for clinical practice [67]. At present, we consider the research foundation for evidence-based recommendations on dosage and route of administration in nausea and vomiting to be very small.

There are a large number of papers dealing with the use of levomepromazine in palliative sedation, most of them recommend its use in combination with midazolam or as a second line drug for continuous sedation if midazolam is ineffective [25,30]–[32,39,42]. Similar data can be found in non-systematic reviews [40,46,68,69]. Again, we could not identify any consensus regarding dosage of levomepromazine for palliative sedation in the included papers; mean doses and dose ranges varied considerably between studies and we found no evidence other than clinical expertise to underpin the choice of dosage and/or level.

In all the above named papers the most common indication for the use of levomepromazine as a sedative agent is in relation to terminal restlessness, especially where this occurs in combination with neuro-psychological symptoms such as confusion, anxiety, agitation or delirium. In the framework for the use of sedation in palliative care published by the European Association for Palliative Care (EAPC, one of the most recent and relevant papers with significant clinical implications), levomepromazine is recommended for sedation of delirious patients as a first line choice, based on the rationale that benzodiazepines, as an initial treatment for delirium, may worsen rather than improve symptoms [7]. However, neither the systematic reviews, nor the studies on palliative sedation, included in this review provide robust evidence, other than clinical expertise for the use of levomepromazine. Papers either provide no information about underlying evidence for recommendations, or recommendations are limited to expert opinions, or findings are based on the same small group of low quality and low evidence studies.

Delirium was considered as a category of its own for the use of levomepromazine in palliative care patients in the present work. Unfortunately, papers that dealt with this indication were scarce and the reported data was highly heterogeneous. The spectrum of data ranged from levomepromazine being ineffective in a case study [58], or the statement that combined treatment of neuroleptics and benzodiazepines are often utilized to control delirium based on data from a retrospective chart review [56] to a systematic review, that included only two studies on levomepromazine, one of which was the case report named above, but nevertheless recommending neuroleptic medications in general as a first or second line pharmacological treatment of delirium [57]. As stated in a work by Caraceni et al. and also mentioned in the EAPC-framework, if control of delirium fails, sedation can be necessary and in these cases levomepromazine may be a choice [7,70]. Thus, it seems that some authors see a smooth transition between treatment of delirium and palliative sedation therapy, but to our knowledge there exist no studies as yet which provide data on dosage levels for delirium versus sedation, or differential co-factors/co-morbidities which would influence the choice of medication, or the meaning of patho-physiological causes of delirium in this context.

A Chochrane review conducted in 2010 about anti-psychotics for acute and chronic pain in adults, proposed levomepromazine for pain within the first 72 hours after acute myocardial infarction [19], and in chronic non-cancer pain management levomepromazine may be used supplementary to other drugs [71]. A couple of studies in the 1960es and 1970es reported levomepromazine to be effective in treatment of pain in cancer patients [72]–[74], and there even seems to be an accepted conversion scale for morphine to levomepromazine of 1.5:1 [75]. Our review included one case report published in 1987, and one study from 1985 underlining these previous findings [41,59], but regrettably there seems to be absolutely no later published research on the use of levomepromazine for pain in palliative care or cancer patients.

Many studies mentioned side effects of levomepromazine, which mainly focused on sedation and hypotension, but skin reactions and extrapyramidal side effects were also reported [13,44,58,59]. Incidences and co-factors of these side effects are not studied in detail, and where such side-effects were reported no specific data for patients at the end of life seems to exist. The above-mentioned Chochrane review on the use of levomepromazine for the treatment of nausea and vomiting will also evaluate associated minor and serious adverse events [67]. Until then it seems that Levomepromazine needs to be considered for use in accordance to expert clinical knowledge and by establishing an indication for its use on ethical considerations, weighing the benefit and harm for an individual patient in clinical practice. Hypotension for example, which is a reported side-effect of Levomepromazine, is unlikely to be a problem in bed-bound patients with a low palliative performance status, and/or a situation in which active symptom control is the only means of providing quality at the end of life [76]. What is more, sedation as a side-effect could be potentially useful and therefore incorporated in a holistic pharmacological regimen of end-of-life/palliative care in some patients.

### Limitations

Because of the limitations of available studies the overall evidence for the use of levomepromazine resulting from the present review remains weak. Findings mainly based on retrospective study designs, lack of control groups, missing randomisation and small sample sizes all lead to a weak level of evidence. More homogeneous prospective studies on larger number of patients, and including measurement and reporting of outcome parameters, should be performed to provide more reliable data.

Our systematic review followed the steps considered good practice including the pre-investigation registration of our review protocol, adherence to the reporting standards and rigorous recording of decision pathways during the review process. However, some limitations apply. We did not perform any meta-analysis as the heterogeneous and low-quality data of the original studies included simply did not allow such a step, and we did not apply risk-of-bias assessment tool across studies. What is more, we limited our review to published data, deliberately excluding grey literature and non-published expert opinion, introducing a publication bias to our review.

## Conclusion

As a consequence of this review we can summarize that there exists some low-grade evidence for the use of levomepromazine for several indications in the palliative care setting. Beneficial effects of levomepromazine are widely reported in the palliative care literature; it’s role in symptom control therefore deserves further evaluation.

Scrutinizing the published literature on levomepromazine it becomes clear, that today there is some low quality and low evidence literature available to support the use of levomepromazine in the palliative care setting. However, only a very few experimental and scientifically sound studies are available. Randomized controlled or even blinded trials on the topic are completely lacking, and although that kind of research may be difficult to manage in the palliative care setting and in every day clinical life [77,78], we should strive for more high quality research. By generating a more solid evidence base for the use of the levomepromazine, its indications, impact and side effects in palliative care, we could gain much needed empirical knowledge for the use of a drug that seems to be clinically effective and multi-factorial in application for end-of-life care. The promise of Levomepromazine as a pharmacological tool, capable of relieving more than one symptom with one dose, exists but needs to be underpinned by further research, and most importantly, studies of experimental design, providing a firmer research base with which to guide future clinical practice.

## Competing interests

All authors declare that they have no competing interests.

## Authors’ contributions

ID and CS carried out the systematic literature review, coordinated the sequence alignment and drafted the manuscript. AS and IL participated in the sequence alignment and in the design of the review and helped to draft the manuscript. All authors read and approved the final manuscript.

## Pre-publication history

The pre-publication history for this paper can be accessed here:

http://www.biomedcentral.com/1472-684X/12/2/prepub

## Supplementary Material

Additional file 1: Appendix 1 Principal characteristics of the included articles. Click here for file

## References

[B1] Klinkenberg M Willems DL van der Wal G Deeg DJ Symptom burden in the last week of life J Pain Symptom Manage 2004 27 1 5 13 10.1016/j.jpainsymman.2003.05.008 14711464

[B2] Laugsand EA Kaasa S de Conno F Hanks G Klepstad P Intensity and treatment of symptoms in 3,030 palliative care patients: a cross-sectional survey of the EAPC Research Network J Opioid Manag 2009 5 1 11 21 19344044 10.5055/jom.2009.0002

[B3] Ventafridda V Ripamonti C De Conno F Tamburini M Cassileth BR Symptom prevalence and control during cancer patients’ last days of life J Palliat Care 1990 6 3 7 11 1700099

[B4] Ellershaw J Ward C Care of the dying patient: the last hours or days of life BMJ 2003 326 7379 30 34 10.1136/bmj.326.7379.30 12511460 PMC1124925

[B5] Ellershaw J Clinical pathways for care of the dying: an innovation to disseminate clinical excellence J Palliat Med 2002 5 4 617 621 10.1089/109662102760269904 12243687

[B6] Fainsinger R Miller MJ Bruera E Hanson J Maceachern T Symptom control during the last week of life on a palliative care unit J Palliat Care 1991 7 1 5 11 2045996

[B7] Cherny NI Radbruch L European Association for Palliative Care (EAPC) recommended framework for the use of sedation in palliative care Palliat Med 2009 23 7 581 593 10.1177/0269216309107024 19858355

[B8] Sivaraman P Rattehalli RD Jayaram MB Levomepromazine for schizophrenia Cochrane Database Syst Rev 2010 10 CD007779 20927765 10.1002/14651858.CD007779.pub2 PMC8973012

[B9] Hals PA Hall H Dahl SG Muscarinic cholinergic and histamine H1 receptor binding of phenothiazine drug metabolites Life Sci 1988 43 5 405 412 10.1016/0024-3205(88)90519-X 2899826

[B10] Lal S Nair NP Cecyre D Quirion R Levomepromazine receptor binding profile in human brain–implications for treatment-resistant schizophrenia Acta Psychiatr Scand 1993 87 6 380 383 10.1111/j.1600-0447.1993.tb03391.x 8395131

[B11] NHS Levomepromazine in Palliative Care http://www.palliativedrugs.com:http://www.palliativecareguidelines.scot.nhs.uk/documents/Levomepromazine.pdf; 2010

[B12] Harris DG Nausea and vomiting in advanced cancer Br Med Bull 2010 96 1 175 185 10.1093/bmb/ldq031 20884654

[B13] Eisenchlas JH Garrigue N Junin M De Simone GG Low-dose levomepromazine in refractory emesis in advanced cancer patients: an open-label study Palliat Med 2005 19 1 71 75 10.1191/0269216305pm972oa 15690871

[B14] Cherny NI Sedation for the care of patients with advanced cancer Nat Clin Pract Oncol 2006 3 9 492 500 16955088 10.1038/ncponc0583

[B15] Palliative cancer care - nausea & vomiting - Management. Levomepromazine (oral or parenteral) http://www.cks.nhs.uk/palliative_cancer_care_nausea_vomiting/management/quick_answers/scenario_nausea_and_vomiting_management/prescriptions/levomepromazine_oral_or_parenteral

[B16] Mannix K Palliation of nausea and vomiting in malignancy Clin Med 2006 6 2 144 147 16688970 10.7861/clinmedicine.6-2-144 PMC4953194

[B17] Klein C Lang U Bukki J Sittl R Ostgathe C Pain management and symptom-oriented drug therapy in palliative care Breast Care 2011 6 1 27 34 10.1159/000324702 21547023 PMC3083268

[B18] Foley KM Controlling cancer pain Hosp Pract 2000 35 4 101 112 10.3810/hp.2000.04.193 10780186

[B19] Seidel S Aigner M Ossege M Pernicka E Wildner B Sycha T Antipsychotics for acute and chronic pain in adults Cochrane Database Syst Rev 2008 4 CD004844 18843669 10.1002/14651858.CD004844.pub2

[B20] Fonzo-Christe C Vukasovic C Wasilewski-Rasca AF Bonnabry P Subcutaneous administration of drugs in the elderly: survey of practice and systematic literature review Palliat Med 2005 19 3 208 219 10.1191/0269216304pm1006oa 15920935

[B21] Sanofi-Aventis Summary of Product Characteristics: Nozinan 2012 http://www.medicinesorguk/emc/medicine/6603/SPC//Nozinan+injection

[B22] Twycross R Wilcock A Palliative Care Formulary 2011 4 palliativedrugs.com

[B23] Davis MP Hallerberg G A systematic review of the treatment of nausea and/or vomiting in cancer unrelated to chemotherapy or radiation J Pain Symptom Manage 2010 39 4 756 767 10.1016/j.jpainsymman.2009.08.010 20413062

[B24] Booth S Moosavi SH Higginson IJ The etiology and management of intractable breathlessness in patients with advanced cancer: a systematic review of pharmacological therapy Nature Clinical Practice Oncology 2008 5 2 90 100 10.1038/ncponc1034 18235441

[B25] Morita T Bito S Kurihara Y Uchitomi Y Development of a clinical guideline for palliative sedation therapy using the Delphi method Journal of Palliative Medicine 2005 8 4 716 729 10.1089/jpm.2005.8.716 16128645

[B26] Liberati A Altman DG Tetzlaff J Mulrow C Gotzsche PC Ioannidis JP Clarke M Devereaux PJ Kleijnen J Moher D The PRISMA statement for reporting systematic reviews and meta-analyses of studies that evaluate health care interventions: explanation and elaboration PLoS medicine 2009 6 7 e1000100 10.1371/journal.pmed.1000100 19621070 PMC2707010

[B27] Sladek R Tieman J Fazekas BS Abernethy AP Currow DC Development of a subject search filter to find information relevant to palliative care in the general medical literature Journal of the Medical Library Association: JMLA 2006 94 4 394 401 17082830 PMC1629429

[B28] Phillips B Ball C Sackett D Badenoch D Straus S Haynes B Dawes M Oxford Centre for Evidence-based Medicine - Levels of Evidence 2011 http://www.pdptoolkit.co.uk/Files/ebm/cebm/Doing%20ebm/levels_of_evidence.htm

[B29] Simera I EQUATOR Network collates resources for good research BMJ 2008 337 a2471 10.1136/bmj.a2471 19001485

[B30] Lebon B Fisher S Case report: Maintaining and withdrawing long-term invasive ventilation in a patient with MND/ALS in a home setting Palliative Medicine 2011 25 3 262 265 10.1177/0269216310389224 21228095

[B31] Alonso-Babarro A Varela-Cerdeira M Torres-Vigil I Rodriguez-Barrientos R Bruera E At-home palliative sedation for end-of-life cancer patients Palliative Medicine 2010 24 5 486 492 10.1177/0269216309359996 20133320

[B32] D’Cruz RS Agar M Seizing the opportunity to consider uncontrollable seizures in the palliative setting Asia-Pacific Journal of Clinical Oncology 2009 5 A243

[B33] Stephenson J The use of sedative drugs at the end of life in a UK hospice Palliative Medicine 2008 22 8 969 970 10.1177/0269216308098801 18952744

[B34] Reuzel RP Hasselaar GJ Vissers KC van der Wilt GJ Groenewoud JM Crul BJ Inappropriateness of using opioids for end-stage palliative sedation: a Dutch study Palliat Med 2008 22 5 641 646 10.1177/0269216308091867 18612030

[B35] Sykes N Thorns A Sedative use in the last week of life and the implications for end-of-life decision making Archives of Internal Medicine 2003 163 3 341 344 10.1001/archinte.163.3.341 12578515

[B36] Gambles M McGlinchey T Dickman A Ellershaw JE Murphy D Agitation and restlessness in the last 24 hours of life on the Liverpool care pathway for the dying patient (LCP) Palliative Medicine 2010 1 S70 10.1136/bmjspcare-2011-000075 24653479

[B37] Morita T Tsunoda J Inoue S Chihara S Effects of high dose opioids and sedatives on survival in terminally ill cancer patients Journal of Pain and Symptom Management 2001 21 4 282 289 10.1016/S0885-3924(01)00258-5 11312042

[B38] Fainsinger RL Waller A Bercovici M Bengston K Landman W Hosking M Nunez-Olarte JM de Moissac D A multicentre international study of sedation for uncontrolled symptoms in terminally ill patients Palliative Medicine 2000 14 4 257 265 10.1191/026921600666097479 10974977

[B39] Chater S Viola R Paterson J Jarvis V Sedation for intractable distress in the dying - A survey of experts Palliative Medicine 1998 12 4 255 269 10.1191/026921698671831786 9743824

[B40] Stone P Phillips C Spruyt O Waight C A comparison of the use of sedatives in a hospital support team and in a hospice Palliat Med 1997 11 2 140 144 10.1177/026921639701100208 9156110

[B41] Oliver DJ The use of methotrimeprazine in terminal care The British journal of clinical practice 1985 39 9 339 340 4063129

[B42] Mercadante S Porzio G Valle A Fusco F Aielli F Costanzo V Palliative sedation in patients with advanced cancer followed at home: a systematic review Journal of Pain and Symptom Management 2011 41 4 754 760 10.1016/j.jpainsymman.2010.07.013 21227633

[B43] De Graeff A Dean M Palliative sedation therapy in the last weeks of life: a literature review and recommendations for standards Journal of Palliative Medicine 2007 10 1 67 85 10.1089/jpm.2006.0139 17298256

[B44] Morita T Chinone Y Ikenaga M Miyoshi M Nakaho T Nishitateno K Sakonji M Shima Y Suenaga K Takigawa C Ethical validity of palliative sedation therapy: a multicenter, prospective, observational study conducted on specialized palliative care units in Japan Journal of Pain and Symptom Management 2005 30 4 308 319 10.1016/j.jpainsymman.2005.03.016 16256895

[B45] Cowan JD Walsh D Terminal sedation in palliative medicine definition and review of the literature Supportive Care in Cancer 2001 9 6 403 407 10.1007/s005200100235 11585266

[B46] Sykes N Thorns A The use of opioids and sedatives at the end of life Lancet Oncology 2003 4 5 312 318 10.1016/S1470-2045(03)01079-9 12732169

[B47] Molassiotis A Brearley SG Stamataki Z Use of antiemetics in the management of chemotherapy-related nausea and vomiting in current UK practice Supportive care in cancer: official journal of the Multinational Association of Supportive Care in Cancer 2011 19 7 949 956 10.1007/s00520-010-0909-7 20574666

[B48] Stephenson J Davies A An assessment of aetiology-based guidelines for the management of nausea and vomiting in patients with advanced cancer Supportive Care in Cancer 2006 14 4 348 353 10.1007/s00520-005-0897-1 16228185

[B49] Amesbury B Alloway L Hickmore E Dewhurst G High-dose levomepromazine (methotrimeprazine) to control nausea in carcinoid syndrome Journal of Palliative Care 2004 20 2 117 118 15332477

[B50] Kennett A Hardy J Shah S A’Hern R An open study of methotrimeprazine in the management of nausea and vomiting in patients with advanced cancer Supportive Care in Cancer 2005 13 9 715 721 10.1007/s00520-004-0768-1 15700129

[B51] Higi M Niederle N Bierbaum W Schmidt CG Seeber S Pronounced antiemetic activity of the antipsychotic drug levomepromacine (L) in patients receiving cancer chemotherapy Journal of cancer research and clinical oncology 1980 97 1 81 86 10.1007/BF00411281 7190568 PMC12253115

[B52] Glare P Pereira G Kristjanson LJ Stockler M Tattersall M Systematic review of the efficacy of antiemetics in the treatment of nausea in patients with far-advanced cancer Supportive Care in Cancer 2004 12 6 432 440 10.1007/s00520-004-0629-y 15108099

[B53] Atkinson CV Kirkham SR Unlicensed uses for medication in a palliative care unit Palliative Medicine 1999 13 2 145 152 10.1191/026921699676057177 10474697

[B54] Douglas C Murtagh FE Chambers EJ Howse M Ellershaw J Symptom management for the adult patient dying with advanced chronic kidney disease: a review of the literature and development of evidence-based guidelines by a United Kingdom Expert Consensus Group Palliat Med 2009 23 2 103 110 10.1177/0269216308100247 19273566

[B55] Twycross R Bankby G Hallowood J The use of low-dose methotrimeprazine (levomepromazine) in the management of nausea and vomiting Prog Palliat Care 1997 5 49 53

[B56] Stiefel F Stagno D Management of insomnia in patients with chronic pain conditions CNS Drugs 2004 18 5 285 296 10.2165/00023210-200418050-00002 15089114

[B57] Kehl KA Treatment of terminal restlessness: A review of the evidence Journal of Pain and Palliative Care Pharmacotherapy 2004 18 1 5 30 15148006

[B58] Fainsinger R Bruera E Treatment of delirium in a terminally ill patient Journal of Pain and Symptom Management 1992 7 1 54 56 10.1016/0885-3924(92)90108-T 1538182

[B59] Rogers AG The use of methotrimeprazine (Levoprome) in a patient sensitive to opioids and possible bowel shutdown J Pain Symptom Manage 1989 4 1 44 45 10.1016/0885-3924(89)90065-1 2703737

[B60] Fletcher AJ Levomepromazine-induced lupus? J Pain Symptom Manage 2009 37 6 10.1016/j.jpainsymman.2008.12.001 19500717

[B61] Harris DG Current Practice in the Management of Terminal Haemorrhage by Palliative Care Teams in the UK Palliat Med (Abstracts of EAPC (European Association for Palliative Care) 2010 24 4 Suppl S5 229 10.1177/0269216310366390 20522880

[B62] Vandenbroucke JP von Elm E Altman DG Gotzsche PC Mulrow CD Pocock SJ Poole C Schlesselman JJ Egger M Strengthening the Reporting of Observational Studies in Epidemiology (STROBE): explanation and elaboration PLoS medicine 2007 4 10 e297 10.1371/journal.pmed.0040297 17941715 PMC2020496

[B63] Sorinola O Olufowobi O Coomarasamy A Khan KS Instructions to authors for case reporting are limited: a review of a core journal list BMC medical education 2004 4 4 10.1186/1472-6920-4-4 15043755 PMC400744

[B64] Kelley K Clark B Brown V Sitzia J Good practice in the conduct and reporting of survey research International journal for quality in health carez: journal of the International Society for Quality in Health Care / ISQua 2003 15 3 261 266 10.1093/intqhc/mzg031 12803354

[B65] Glare PA Dunwoodie D Clark K Ward A Yates P Ryan S Hardy JR Treatment of nausea and vomiting in terminally ill cancer patients Drugs 2008 68 18 2575 2590 10.2165/0003495-200868180-00004 19093700

[B66] Glare P Miller J Nikolova T Tickoo R Treating nausea and vomiting in palliative care: a review Clinical interventions in aging 2011 6 243 259 21966219 10.2147/CIA.S13109 PMC3180521

[B67] Darvill E Dorman S Perkins P Levomepromazine for nausea and vomiting in palliative care 2012 In: Cochrane Protocol. Cochrane Database 10.1002/14651858.CD009420.pub2 23633372

[B68] Adam J ABC of palliative care. The last 48 hours British Medical Journal 1997 315 7122 1600 1603 10.1136/bmj.315.7122.1600 9437282 PMC2127988

[B69] Vissers KCP Hasselaar J Verhagen SAHHVM Sedation in palliative care Current Opinion in Anaesthesiology 2007 20 2 137 142 10.1097/ACO.0b013e328049557b 17413397

[B70] Caraceni A Simonetti F Palliating delirium in patients with cancer The Lancet Oncology 2009 10 2 164 172 10.1016/S1470-2045(09)70018-X 19185834

[B71] Merskey H Pharmacological approaches other than opioids in chronic non-cancer pain management Acta anaesthesiologica Scandinavica 1997 41 1 Pt 2 187 190 9061105 10.1111/j.1399-6576.1997.tb04636.x

[B72] Lasagna L Dekornfeld TJ Methotrimeprazine: a new phenothiazine derivative with analgesic properties JAMA: the journal of the American Medical Association 1961 178 887 890 10.1001/jama.1961.03040480017004 14462565

[B73] Beaver WT Wallenstein SL Houde RW Rogers A A comparison of the analgesic effects of methotrimeprazine and morphine in patients with cancer Clinical pharmacology and therapeutics 1966 7 4 436 446 5328470 10.1002/cpt196674436

[B74] Davidsen O Lindeneg O Walsh M Analgesic treatment with levomepromazine in acute myocardial infarction A randomized clinical trial. Acta medica Scandinavica 1979 205 3 191 194 10.1111/j.0954-6820.1979.tb06029.x 371339

[B75] Bloomfield S Simard-Savoie S Bernier J Tetreault L Comparative Analgesic Activity of Levomepromazine and Morphine in Patients with Chronic Pain Canadian Medical Association journal 1964 90 1156 1159 14145469 PMC1922768

[B76] Stone P Rees E Hardy JR End of life care in patients with malignant disease Eur J Cancer 2001 37 9 1070 1075 10.1016/S0959-8049(01)00087-9 11378335

[B77] Grande GE Todd CJ Why are trials in palliative care so difficult? Palliat Med 2000 14 1 69 74 10.1191/026921600677940614 10717728

[B78] Hudson P Aranda S McMurray N Randomized controlled trials in palliative care: overcoming the obstacles International journal of palliative nursing 2001 7 9 427 434 11832846 10.12968/ijpn.2001.7.9.9301

