# Supplementary material for: Evidence for the use of Levomepromazine for symptom control in the palliative care setting: a systematic review
Source: BMC Palliat Care. 2013 Jan 19;12:2. doi: 10.1186/1472-684X-12-2 (PMC3602665; doi:10.1186/1472-684X-12-2)
Supplement: Additional file 1: Appendix 1 — Principal characteristics of the included articles. [file 1472-684X-12-2-S1.docx]

| **author** | **year** | **study design** | **principal indication** | **population** | **number of study participants** | **number of patients under treatment (levome-promazine)** | **results** | **quality checklists** | **level of evidence** |
| --- | --- | --- | --- | --- | --- | --- | --- | --- | --- |
| Higi[[51](#_ENREF_51)] | 1980 | prospective observational | nausea | cancer | 113 | 113 (100%) | - most pronounced effect with levomepromazine was seen in patients treated with 20 mg daily x 5 schedule of cisplatin but complete or partial relief was also seen with high single cisplatin doses of 50-100 mg | STROBE 9/22 | 3b |
| Oliver[[41](#_ENREF_41)] | 1985 | retrospective chart review | several | cancer | 675 | 80 (12%) | - levomepromazine is an effective drug in the control of some difficult symptoms in the terminal care of cancer patients - main indications for levo: confusion and agitation, vomiting and pain | STROBE 12/22 | 3b |
| Rogers[[59](#_ENREF_59)] | 1987 | case report | pain | lung cancer | 1 | 1 | - in cases of sensitivity to many opioids and the present possibility of a bowel shutdown, levomepromazine can be an appropriate drug - levomepromazine has little effect on the gut, less respiratory depressant effect than an equianalgesic dose of morphine, and is an effective antiemetic - 10 mg i.m are approximately equivalent to morphine 5 mg i.m. | Sorinola: 3/9 | 4 |
| Fainsinger [[58](#_ENREF_58)] | 1992 | case report | delirium | carcinoma of the prostate | 1 | 1 | - in agitated delirium levomepromazine was given after haloperidol and lorazepam but the patient did not respond and the presence of extrapyramidal side effects contributed to the decision to change treatment - midazolam finally controlled delirium | Sorinola: 5/9 | 4 |
| Stiefel[[56](#_ENREF_56)] | 1992 | retrospective chart review | delirium | cancer | 39 | 7 (18%) | - a combined treatment of neuroleptics and benzodiazepines is often utilized to control delirium and alleviate the side effects of neuroleptics | STROBE 7/22 | 3b |
| Stone[[40](#_ENREF_40)] | 1997 | retrospective chart review | sedation | not defined | 115 | 45 (39%) | - sedatives are used for symptom control more frequently in the hospice than in the hospital | STROBE 9/22 | 3b |
| Chater[[39](#_ENREF_39)] | 1998 | survey study | sedation | not defined | 100 | 30 (30%) | - after midazolam levomepromazine was the second most commonly used drug for palliative sedation | Kelley: 5/7 | 4 |
| Atkinson[[53](#_ENREF_53)] | 1999 | prospective observational | others (unlicensed indications) | cancer | 76 | 8 (10%) | - levomepromazine is listed for use within palliative care specifically and is licensed for the management of nausea, vomiting and sedation | STROBE 12/22 | 3b |
| Fainsinger[[38](#_ENREF_38)] | 2000 | prospective observational, multicenter | sedation | mixed | 387 | 1 (0,26%) | - midazolam was the most common medication prescribed for sedation | STROBE 11/22 | 3b |
| Cowan[[45](#_ENREF_45)] | 2001 | systematic review | sedation | not defined | 790* | 125* (16%) | - most often midazolam was used for terminal sedation | PRISMA 10/27 | 3a |
| Morita[[37](#_ENREF_37)] | 2001 | prospective | sedation/ survival | cancer | 209 | 2 (1%) | - use of strong sedatives showed no significant influence on patient survival | STROBE 20/22 | 3b |
| Sykes[[35](#_ENREF_35)] | 2003 | retrospective case-control | sedation | mixed | 237 (114 receiving sedation) | 51 (45% of sedated patients, 22% of the total) | - 30 patients received levomepromazine on the last day of their life but only 3 patients received sedative doses of levomepromazine throughout their last week of life - 22 patients received levo in their last 7 days - levomepromazine can be used as sedative at the end of life - most patients received additional potentially sedative doses of midazolam. | STROBE 18/22 | 3b |
| Glare[[52](#_ENREF_52)] | 2004 | systematic review | nausea | cancer | 29*° | 29*° (100%) | - there was little or no evidence of the efficacy of methotrimeprazine in nausea and vomiting | PRISMA 12/27 | 3a |
| Amesbury[[49](#_ENREF_49)] | 2004 | case report | nausea | carcinoid tumor | 1 | 1 | - because of its 5HT2 antagonist property levomepromazine is a logical choice as antiemetic in case of carcinoid syndrome, when large amounts of circulating 5-HT are present | Sorinola: 6/9 | 4 |
| Kennett[[50](#_ENREF_50)] | 2004 | prospective observational | nausea | mixed | 65 | 65 (100%) | - levomepromazine has antiemetic activity even in those patients who have failed previous antiemetic therapy - it is a logical choice for patients in whom the exact cause of nausea and vomiting is unknown or in whom other antiemetics have failed | STROBE 20/22 | 2b |
| Kehl[[57](#_ENREF_57)] | 2004 | systematic review | restlessness | not defined | no data | no data | - neuroleptic medications is a first or second line pharmacological treatment of restlessness | PRISMA 12/27 | 3a |
| Stephenson[[48](#_ENREF_48)] | 2005 | prospective observational | nausea | cancer | 121 | 27 (22%) | - an approach using aetiology-based guidelines in the management of nausea and vomiting is moderately effective, although there are some patients refractory to standard antiemetic regimens - levomepromazine is first line in indeterminate causes and second line in all other causes | STROBE 12/22 | 3b |
| Eisenchlas [[13](#_ENREF_13)] | 2005 | prospective open-label | nausea | digestive cancer | 70 | 70 (100%) | - treatment with low-dose levomepromazine is an effective and safe option for advanced cancer patients who fail to respond to first-line antiemetic treatment - Pearson test revealed no association between levomepromazine dose and response to treatment and no association between levomepromazine dose and degree of sedation | STROBE 18/22 | 2b |
| Morita T[[25](#_ENREF_25)] | 2005 | systematic review | sedation | not defined | no data | no data | - levomepromazine is used in continuous sedation as first choice if midazolam is ineffective. | PRISMA 4/27 | 3a |
| DeGraeff[[43](#_ENREF_43)] | 2007 | systematic review | sedation | not defined | 1127* | 206* (18%) | - levomepromzine is the most commonly used antipsychotic drug for sedation, often given in conjunction with benzodiazepines - sedation for delirium should only be considered after adequate treatment with haloperidol or other antipsychotics - in refractory cases treatment with midazolam + haloperidol or levomepromazine should be considered | PRISMA 7/27 | 3a |
| Booth S[[24](#_ENREF_24)] | 2008 | systematic review | breathlessness | cancer | no data | no data | - no randomized controlled trials of phenothiazines for treatment of breathlessness in patients with cancer exists - levomepromazine or haloperidol is recommended fear, rather than anxiety, becomes overwhelming or for sedation at the end of life | PRISMA 2/27 | 5 |
| Stephenson[[33](#_ENREF_33)] | 2008 | retrospective chart review | sedation | not defined | 156 (1996), 194 (2006) | 80 (51%, 1996), 112 (58%, 2006) | - levomepromazine was often used first line for sedation in 1996, whereas 2006 midazolam is used first line and levomepromazine as an adjunct | STROBE 6/22 | 3b |
| Reuzel[[34](#_ENREF_34)] | 2008 | retrospective survey | sedation | not defined | 312 | 15 (5%) | - opioids alone were administered for 22% of palliative sedation | Kelley: 6/7 | 3b |
| Fletcher[[60](#_ENREF_60)] | 2008 | case report | side effect | metastatic non-small cell lung cancer, | 1 | 1 | - symptoms of drug-induced lupus secondary to levomepromazine may occur after months of treatment with levomepromazine and resolving within days of discontinuing it - as levomepromazine is increasingly used in the palliative care setting, it is important to be aware of its side-effect profile | Sorinola: 7/9 | 4 |
| D'Cruz[[32](#_ENREF_32)] | 2009 | case report | sedation | metastatic insular thyroid cancer | 1 | 1 | - subcutaneous infusion was commenced with midazolam as anti-convulsant and levomepromazine for agitation/delirium was successful in controlling her seizures but the effect can not clearly be ascriptioned to levomepromazine | Sorinola: 3/9 | 4 |
| Douglas[[54](#_ENREF_54)] | 2009 | systematic review | other (symptoms in chronic kidney disease) | chronic kidney disease | no data | no data | - if nausea and vomiting in the patient dying with advanced chronic kidney disease persist after haloperidol, levomepromazine is an alternative antiemetic - it is a second choice for terminal agitation added to midazolam | PRISMA 10/27 | 3a |
| Davis[[23](#_ENREF_23)] | 2010 | systematic review | nausea | cancer | 136*° | 136*° (100%) | - levomepromazine is not mentioned in expert opinion guidelines; least low-quality RCTs or multiple prospective studies have demonstrated responses in nausea and vomiting - levomepromazine may be an effective antiemetic in those with advanced carcinoid | PRISMA 9/27 | 3a |
| Molassiotis [[47](#_ENREF_47)] | 2010 | survey study | nausea (chemotherapy-related) | cancer | 154 | no data | - levomepromazine was second-/third-line option for refractory chemotherapy-related nausea and vomiting which failed to respond to domperidone, metoclopramide and cyclizine - levomepromazine is one of the most common rescue antimetics | Kelley: 7/7 | 3b |
| Lebon[[30](#_ENREF_30)] | 2010 | case report | sedation | motorneuron disease | 1 | 1 | - if in case of ALS and withdrawal from mechanical ventilation sedation with midazolam is insufficient, levomepromazine can be given additional | Sorinola: 7/9 | 4 |
| Alonso-Babarro[[31](#_ENREF_31)] | 2010 | retrospective cohort study | sedation | cancer | 29 | 2 (7%) | - two patients needed to have their PS medication changed to levomepromazine, which was effective in both patients - palliative sedation checklists recommends beginning with midazolam followed by levomepromazine if midazolam is ineffective | STROBE 10/22 | 2b |
| Gambles[[36](#_ENREF_36)] | 2010 | retrospective study | sedation | not defined | 3893 | no data | - only around half of patients received medication for agitation and restlessness in the last 24 hours of life - the total amount given was low suggesting care was tailored to individual need for those patients whose care was supported by the LCP | STROBE: 4/22 | 3b |
| Harris[[61](#_ENREF_61)] | 2010 | retrospective survey | several | cancer | 155 | no data | - midazolam was the most commonly used crisis medication, levomepromazine was also used | Kelley: 4/7 | 3b |
| Mercadante[[42](#_ENREF_42)] | 2011 | systematic review | sedation | cancer | 29* | 2* (7%) | - benzodiazepines, specifically midazolam, have been most frequently used alone or in combination with neuroleptics for sedation | PRISMA 10/27 | 3a |
